# Replacing Fish Meal with Defatted Insect Meal (Yellow Mealworm *Tenebrio molitor*) Improves the Growth and Immunity of Pacific White Shrimp (*Litopenaeus vannamei*)

**DOI:** 10.3390/ani9050258

**Published:** 2019-05-21

**Authors:** Constant Motte, Alfredo Rios, Thomas Lefebvre, Hong Do, Morgane Henry, Orapint Jintasataporn

**Affiliations:** 1 Ÿnsect, R&D Department, Genopole, 91058 Evry, France; tle@ynsect.com (T.L.); hdo@ynsect.com (H.D.); 2 Hellenic Centre for Marine Research, Institute of Molecular Biology, Biotechnology and Aquaculture, Agios Kosmas, 16777 Helliniko, Greece; morgane@hcmr.gr; 3 Department of Aquaculture, Faculty of Fisheries, Kasetsart University, Bangkok 10900, Thailand; ffisora@ku.ac.th

**Keywords:** insect meal, *Tenebrio molitor*, yellow mealworm, fish meal replacement, *Litopenaeus vannamei*, growth performances, FCR, immunity, *Vibrio parahaemolyticus*

## Abstract

**Simple Summary:**

Globally, Pacific white shrimp is one of the most commonly commercialized marine species in aquaculture. In recent years, the commercial production of this shrimp has experienced major challenges, including the availability and cost of fish meal—the main protein source of commercial shrimp—as well as frequent outbreaks of early mortality syndrome (EMS) among shrimp stock. In the present study, we investigated whether substituting fish meal with a defatted yellow mealworm meal could overcome these challenges. Our results from a series of feeding trials show that it is possible to partially or completely replace fish meal with this insect meal in isoproteic and isoenergetic diets. The insect meal improved growth and feed conversion performances of shrimp; optimum performances were achieved at 50% of fish meal replacement. Furthermore, shrimp that were fed the insect meal and then challenged with the pathogenic bacteria that cause EMS (*Vibrio parahaemolyticus*) had significantly improved survival rates and reduced immunosuppression. We conclude that an insect meal comprised of *Tenebrio molitor* mealworm, is a suitable alternative to fish meal in the commercial production of shrimp because of the meal’s high protein value and the presence of chitin/other bioactive substances that counter pathogen infection.

**Abstract:**

Recently, ecological and economic issues have affected fish meal (FM) supply, the main source of protein for shrimp. This triggered a search for alternative dietary protein sources for shrimp production. We studied the consequences of replacing FM with a defatted insect meal, ŸnMeal^TM^ (YM), comprised of yellow mealworm (*Tenebrio molitor*). Growth and immune parameters of juvenile Pacific white shrimp (*Litopenaeus vannanmei*) were compared after an eight-week feeding trial. Shrimp were kept in aquaria with densities of 60 and 40 shrimp/m^2^ and fed one of five diets in which a proportion of FM was replaced by YM. All diets were isoproteic, isoenergetic, and balanced in lysine and methionine. After the feeding trial, shrimp were challenged with pathogenic bacteria (*Vibrio parahaemolyticus*). Growth and feed conversion parameters improved when YM was included in shrimp diets; with the highest weight gain and best food conversion ratio (FCR) achieved when 50% of FM was replaced by YM versus the control diet that contained no YM (initial weight: 1.60 g/shrimp; growth: 5.27 vs. 3.94 g/shrimp; FCR 1.20 vs. 1.59). In challenged shrimp, mortality rates were significantly less among groups that received YM, with a 76.9% lower mortality rate in the 50% FM replacement group versus the control.

## 1. Introduction

Pacific white shrimp (*Litopenaeus [L.] vannamei)*, is one of the most commonly produced shrimp species in the world with production levels reported to be more than 3.67 million metric tons in 2014 [1]. Fish meal (FM) is a very important and significant protein source in aquaculture [2]. Recently, a combination of ecological problems and economic issues have affected FM supply and this, in turn, has triggered a search for alternative dietary protein sources, such as by-products or plant proteins [3,4,5,6]. FM contains the essential amino acids, minerals, and nucleotides needed for commercially produced carnivorous aquaculture species and FM is, therefore, an essential component in the diets of fish and shrimp to maintain production, growth, and health in general [7].

According to the Food and Agriculture Organization (FAO) of the United Nations, insects can be a sustainable source of proteins. Insect meal provides a good source of amino acids, lipids, minerals, vitamins, and energy [7,8,9,10]. The larval stage of the yellow mealworm, *Tenebrio molitor*, is a good candidate to be used as a high protein feed ingredient that could replace FM for carnivorous aquaculture species [11,12,13,14,15]. Globally, the insect industry is growing fast. The International Platform of Insects for Food and Feed estimates the current insect production at 6000 metric tons, which represents an annual global investment of 355 million euro [16].

Studies evaluating the replacement of FM with full-fat insect meal for commercially produced shrimp and fish, have reported dissimilar growth performances within the same species. This may be a consequence of differences in the meal preparation process and/or the formulation of diets. The nutritional quality of animal protein meal is directly linked to the processing and the freshness of the raw material used [17]. For instance, in rainbow trout, a study showed that a black soldier fly larval meal could replace 50% of FM without affecting growth. However, a protein utilization efficiency reduction was observed [18]. Concerning shrimp, Cummins et al. (2017) reported that a full-fat black soldier fly (*Hermetia illucens*) meal can replace up to 25% of FM without affecting shrimp growth [19]. Similarly, previous studies on *T. molitor* full-fat meal-based diets that were adequately supplemented with methionine, demonstrated similar or superior nutritional value and growth performance of Pacific white shrimp compared to the FM-based diet [20,21].

Other factors affecting shrimp production are disease outbreaks which are considered to be linked to diminished growth rates and high mortalities, especially in the context of the pathogenic species of *Vibrio* spp. in pond waters of shrimp farms [22,23]. The acute hepatopancreatic necrosis disease caused by the pathogen *Vibrio parahaemolyticus* on shrimp has, over the past decades, resulted in large losses in production [24]. Shrimp do not have an adaptive immune system to fight diseases [25]. Thus, in the context of intensive shrimp farming, producers have to find ways to boost the innate immune system of shrimp to improve disease resistance [25]. Consequently, the health of shrimp and the enhancement of its innate immune system are of primary concern. Dietary chitin and krill (chitin-rich) have been shown to modulate the immune system of fish and shrimp [26,27]. In contrast with FM, insect meals contain chitin, which is a b-1,4-linked polymer of N-acetyl-d-glucosamine, one of the most abundant polysaccharides in nature, and a common constituent of insect exoskeleton, crustacean shells, and fungal cell walls [28].

Currently, the aquaculture industry is looking for alternative proteins sources for commercial shrimp rearing—proteins that are good candidates to maintain positive growth performance as well as improve the immunity and disease resistance of shrimp. Hence, insect meals may play an important role in shrimp farming economics and sustainability compares to both fish meal and plant meal-based diets. Indeed, the insect environmental footprint has been shown to be much lower than plant proteins as their production requires less arable land, water, and energy [8,10].

The goal of the present study was to evaluate the dietary effects of a processed and defatted yellow mealworm (*T. molitor*) meal on juvenile shrimp. The study also evaluated the immune response and resistance to a common bacterial pathogen, *V. parahaemolyticus*.

## 2. Materials and Methods

### 2.1. Shrimp and Experimental Conditions

The experiments were carried out at the Nutrition and Aquafeed Laboratory, Department of Aquaculture, Faculty of Fisheries, Kasetsart University, Thailand. Post larvae of 0.5 g (PL-25) of the Pacific white shrimp (*L. vannamei*) issued from a specific pathogen-free lineage were obtained from a nursery in the Samutsakhon Province, Thailand. After 21 days of acclimation, juvenile shrimp of 1.5–1.6 g were randomly distributed into 100 L aquaria. The stocking density was fixed at 60 shrimp/m^2^ (15 individuals/aquarium). These aquaria contained 80 L of 15 ppt saline water with a pH ranging from 7.0–7.5 and total ammonia nitrogen below 0.02 mg/L NH3-N. Oxygen was supplied by a blower in each tank and kept at concentrations higher than 5 mg/L. Feedings were supplied 3 times a day at 3–5% of shrimp body weight for 8 weeks. Unconsumed feed and feces were siphoned daily, and 20% of the water was exchanged every 3 days. Temperature ranged between 26 and 30 °C. Each treatment was replicated 6 times.

### 2.2. Experimental Design and Diet Preparation

#### 2.2.1. Yellow Mealworm Meal Composition

The yellow mealworm meal was provided by Ÿnsect (Damparis, France). It is a commercial product named ŸnMeal^TM^ (YM), which is a dried powder obtained by processing larvae of *T. molitor* reared on vegetal feed material. YM is defatted and the proximate composition of this insect meal is detailed in Table 1.

#### 2.2.2. Diet Preparation

Five experimental diets were designed to gradually reduce the proportion of FM, while increasing the content of YM. The FM was replaced at the following levels: 0% (control), 25%, 50%, 75%, and 100% (complete replacement) by YM while soy oil and wheat flour content were adjusted. All diets were formulated to be isoproteic and isolipidic, respecting the values of lysine and methionine according to the NRC 2011 recommendations [29]. The feed formulation and proximate composition of the diets are detailed in Table 2.

Feed materials were ground to 150–250 micron, mixed together, and then gently humidified to reach 25% of moisture. The resulting wet mixture was passed through a Hobart mincer to form 2 × 2 mm (diameter and length) pellets. Diets were air-dried at 65 °C for 12 h to reach approximately 10% moisture. Dry pellets were finally stored at −20 °C in vacuum-packed and hermetically sealed plastic bags.

### 2.3. Growth and Feed Conversion Assessment

During the experimental period, the amounts of supplied feed were recorded, and unconsumed feed was weighted after siphoning, filtering, and drying. These data were used to calculate the feed consumed. Shrimp were sampled biweekly until the end of the 8 weeks for growth assessment. Mortality was monitored daily. Growth performances were estimated through the assessment of weight gain, average daily weight gain, specific growth rate (SGR), and mortality rate. Feed use performances were estimated by feed conversion (FCR) and protein efficiency (PER) ratios.

(1) $$\mathrm{WG}\;=\;W_{t}-W_{0}$$

(2) $$\mathrm{ADG}\;\left(g/d\right)=\frac{W_{t}-W_{0}}{rearing\;days}$$

(3) $$\mathrm{SGR}\;\left(\%/d\right)=100\times \;\frac{\ln(W_{t})\;-\;\ln\left(W_{0}\right)}{t\;\left(rearing\;days\right)}$$

(4) $$\mathrm{FCR}=\;\frac{\mathrm{Total}\;\mathrm{dry}\;\mathrm{feed}\;\mathrm{intake}\;}{W_{t}-W_{0}}$$

(5) $$\mathrm{PER}=\frac{W_{t}-W_{0}}{\mathrm{Total}\;\mathrm{feed}\;\mathrm{intake}\;x\;\mathrm{Protein}\;\mathrm{content}\;\mathrm{of}\;\mathrm{the}\;\mathrm{diet}}$$

where *W_t_* is the weight per shrimp per tank obtained at time *t*; WG: weight gain; ADG: average daily gain; PER: protein efficiency ratio.

### 2.4. Challenge Test Using Vibrio Parahaemolyticus

The resistance of Pacific white shrimp against *V. parahaemolyticus* was assessed immediately after the 8-week feeding trial. The pathogenic strain was supplied by the Coastal Aquaculture Research and Development Regional Center 2 (Samutsakhorn), Department of Fisheries, Ministry of Agriculture and Cooperative, Thailand. Three replicates of 10 shrimp per treatment were randomly sampled from the feeding trial and used for the challenge test. The challenge test was performed by infecting each shrimp with an intramuscular injection of 4.3 × 10^5^ CFU/mL of *V. parahaemolyticus*. The same number of shrimp per treatment was also randomly sampled then injected with saline (0.85%) to be used as control. Experimental shrimp were kept in the same aquaria and rearing condition as described previously. Each treatment was replicated 3 times. Mortality was monitored for 10 days.

### 2.5. Immune Parameters

The immunity status of shrimp was evaluated at the end of feeding trial and 16 h after the challenge with *V. parahaemolyticus* by measuring the total hemocyte count (THC), hemolymph protein level (HPL), phenoloxidase (PO) activity, and clearance ability. Nine shrimp were harvested from each treatment, 3 individuals from each replicate, to obtain hemolymph samples. These hemolymph samples were withdrawn individually from the base of the third walking leg using a syringe containing an anticoagulant (KC-199 medium plus HEPES 2.38 g/L, supplemented with 5% L-cysteine, Itami et al., 1994 [30]). The resulting mixture of hemolymph and anticoagulant was used to measure the immune parameters (THC, HPL, and PO) as described below.

#### 2.5.1. Total Hemocyte Count

Hemolymph 0.1 mL from three randomly selected shrimp per treatment were mix with 0.5 mL modified KC-199 medium (Itami et al., 1994 [30]) before the addition of trypan blue and subsequent gentle mixing. Live hemocytes were counted and calculated as cells/mL using a hemocytometer under light microscopy at 400 × magnification.

#### 2.5.2. Phenoloxidase Activity Assay

PO activity was assayed spectrophotometrically using L-3, 4-dihydroxyphenylalanine (L-DOPA; Sigma, Saint Louis, MO, USA) as substrate and trypsin (Sigma, cat. no. T0646) as elicitor following the method described by Smith and Soderhall (1991) [31]. Shrimp hemolymph 0.1 mL was mixed with 0.4 mL modified KC-199 medium anticoagulant, then centrifuged at 2500 rpm for 10 min at 4 °C. Hemocytes were washed and collected in ice-cold cacodylate (CAC) buffer at pH 7.0. The hemocyte lysate supernatant (HLS) was prepared using a sonicator at a microtip output 5, duty cycle 50% for 3 s, before centrifugation at 15,000 rpm for 20 min at 4 °C. The supernatant (HLS) was used as an enzyme source for the assay. PO activity assay was conducted on a total of 200 µL HLS incubated with 200 µL of 0.1% trypsin in CAC buffer at room temperature for 30 min, and mixed with 200 µL of L-DOPA 0.3% in CAC buffer. Each reaction mixture was further diluted with 600 µL of CAC buffer then mixed and optical density was measured at a 490 nm. Absorbance measurements were made against a blank consisting of CAC buffer, L-DOPA, and elicitor to control for spontaneous oxidation of the substrate alone. One unit of enzyme activity was defined as an increase in absorbance of 0.001/min/mg protein.

#### 2.5.3. Hemolymph Protein

Hemolymph protein content in HLS was measured by the Lowry method using bovine serum albumin as a standard protein (Lowry, et al., 1951) [32].

#### 2.5.4. Clearance Ability

At the end of the feeding trial, the ability of each shrimp to remove bacterial cells from their hemolymph circulation system was measured by the modified method of Martin et al. (1993) [33]. A bacterial suspension of *V. parahaemolyticus* of 0.1 mL (4.3 × 10^5^ cfu/mL; 4.6 log cfu/mL) was injected into the tail muscle of each shrimp and then kept in the aquaria with sea water for 3 h. Hemolymph was collected from each shrimp and then 30 µL of whole blood was dropped on TCBS agar and a two-fold dilution of whole blood was made using sterile 2.6% NaCl solution. The number of bacteria was counted on the TCBS above and reported in log cfu/mL.

### 2.6. Statistical Analysis

Experimental units in all bioassays were attributed to each treatment in a complete randomized experimental design. All statistical analyses were performed with the statistical software R (version 3.5.3). Polynomial contrasts were used to test linear, quadratic, and cubic trends between the different growth responses and %FM replacement. If an ANOVA’s resulting from these analyses was significant, least square means (R: lsmeans function) were used to test pairwise differences among FM replacement levels. Pairwise comparisons used Tukey’s honest significant difference test. Statistical tests were considered significant at an alpha = 0.05. Residuals were checked for normality using quantile to quantile plots and Bartlett tests were used to assess the homogeneity of variances. Generalized linear models with a binomial error distribution (R: function glm) were used to detect differences in mortality among %FM fishmeal replacement in the growth and challenge assays using the Likelihood-ratio test. Pairwise treatment differences were assessed with Tukey’s honest significant difference test.

Generalized least squares regression (R: gls function) was used to model the different immunological responses as a function of FM replacement, challenge status (‘pre-challenged’ or challenged), and their interaction. This interaction term was used to test differences in the response slopes between ‘pre-challenge’ and bacteria challenged shrimp. A compound symmetry correlation structure was specified in the model to account for the dependence of measurements (‘pre-challenge’ vs. challenged) made on the same group of shrimp. Whenever non-homogeneity of variances between the different shrimp status was observed, individual variances were fitted to each status group. Similarly, the Wald Chi-square test was used to test the significance of estimated parameters in the models.

## 3. Results

### 3.1. Growth and Feed Conversion Performances of Pacific White Shrimp Fed a Yellow Mealworm Diet as Replacement of Fish Meal

The analyses showed significant quadratic responses to FM replacement in both final biomass and body weight (*p* = 0.005 and *p* = 0.004, respectively, Table 3). The largest final biomass and body weights were achieved with a diet in which 50% FM was replaced by YM; an increase of 24% in body weight when compared with the control group (0% FM replacement, Table 3) Similarly, a significant quadratic response to FM replacement was observed in weight gain (*p* = 0.005). The largest weight gain difference (34%) was observed in the 50% FM replacement diet group versus the control group. SGR showed a cubic response (*p* = 0.0272); there was a trend towards an increase in SGR in the group that received a 25% FM replacement diet versus the control. The SGR for groups that received 50% and 100% FM replacement diets, respectively, were significantly higher than that of the control although the SGR in these groups did not differ significantly from one another. We observed no significant differences in shrimp mortality among the diet groups.

#### Feed Utilization

At the end of the 8-week feeding trial, there were no significant differences in the average cumulative feed intake between dietary groups. The mean total feed intake for all dietary groups amounted to 6.31 gram/shrimp. A FCR quadratic trend (*p* = 0.02) was detected among the diet groups. The 50% FM replacement diet had a FCR that was 24% lower than that of the control group. Similarly, a significant quadratic response was observed in protein efficiency ratios with the highest value at 50% FM replacement.

### 3.2. Mortality and Immunity Following the Challenge Tests with Vibrio Parahaemolyticus

#### 3.2.1. Mortality Rate

**Figure 1:**
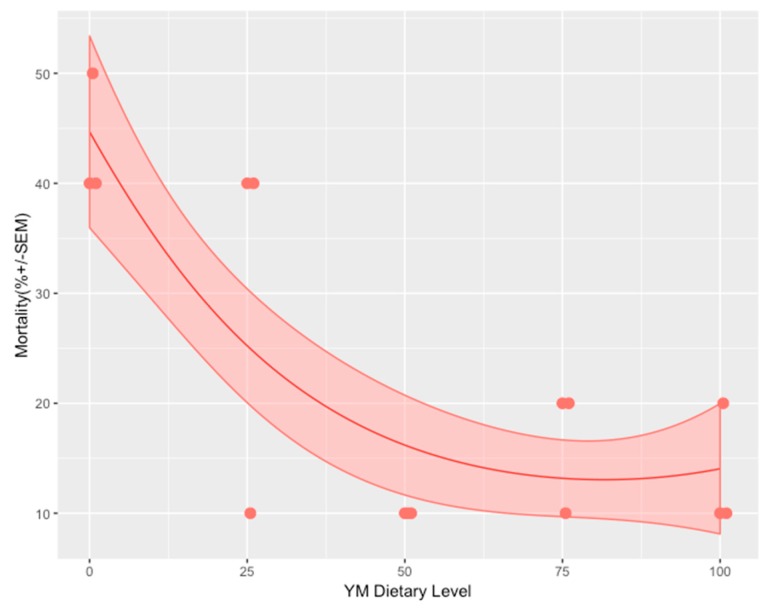
Cumulative mortality as a function of ŸnMeal^TM^ (YM) dietary level on Day 10 after immune challenge with *Vibrio parahaemolyticus* (n = 30).

A generalized linear regression model with a binomial error distribution showed a significant decreasing slope in mortality rate as a function of FM replacement (*p* = 0.032). This slope started to plateau from the 50% FM replacement (Figure 1 and Table 4). All dietary groups, except for the group that received the 25% FM replacement diet, showed significantly different mortality rates when compared to the FM control group. In the experiment, the mortality rate was more than four times higher in the control group than in the group that received the 50% FM replacement.

#### 3.2.2. Immunity Status

**Figure 2:**
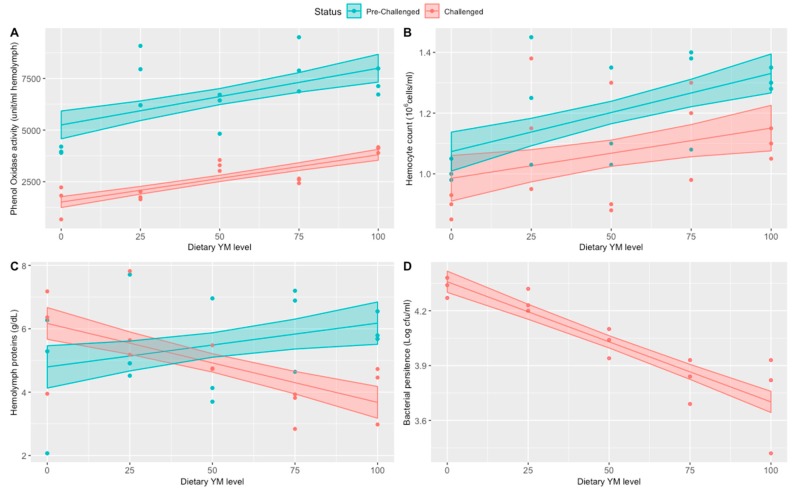
Immune status of the shrimp before and after bacterial challenge. (**A**) Phenoloxidase (**B**) Total hemocyte counts (**C**) hemolymph protein (**D**) persistent bacterial numbers in hemolymph 3 h after the bacterial challenge. Confidence bands for each fitted line correspond to one SEM (n = 3).

The FM replacement and the bacterial challenge both had independent and interaction effects on the immune system of the shrimps (Figure 2, Supplementary Material Figure S2, and Table 5). All three immunological parameters (PO, THC, and TP) tested on the pre-challenged shrimp correlated positively with the dietary YM levels. On the challenged shrimp, both and THC positively correlated with the YM levels whereas protein hemolymph levels and bacterial persistence in hemolymph decreased with increasing levels of dietary YM.

Concerning the PO activity following bacterial inoculation, there was a significant decrease in PO activity (60%, *p* < 0.0001) in all shrimp groups when compared with the PO levels in pre-challenged shrimp (Figure 2A, Table 5). In bacterial challenged shrimp, PO levels were 2.5 times higher (*p* < 0.0001) in the group that received 100% YM diet versus the 100% FM control group. No significant interaction (*p* = 0.735) was found between challenge status and dietary YM level (Table 5).

The model for THC showed a significant interaction (*p* = 0.0084) between shrimp challenge status and dietary YM level, indicating a lower intercept and rate of THC increase as a function of dietary YM for the challenged shrimp versus the pre-challenged ones. The fitted line for challenged shrimp was lower than that for pre-challenged shrimp, indicating a significant suppression of THC by the bacterial infection (Figure 2B and Table 5), but with a similar trend across different levels of YM inclusion).

Similarly, the fitted model for hemolymph protein showed an interaction (*p* = 2.2 × 10^−5^), which indicated two different linear trends in the data; positive and negative trends between hemolymph protein and dietary YM level for pre-challenged and challenged shrimp, respectively (Figure 2C and Table 5).

Finally, a linear regression indicated a steep downward trend between the log of colony forming units per ml and the dietary YM level (Figure 2D and Table 5).

## 4. Discussion

The main objective of the present study was to evaluate whether FM can be replaced by YM in the diet of shrimp reared in commercial operations. We assessed the growth and feed use performance as well as immune parameters of shrimp that were fed diets which contained different ratios of YM and FM. Because the growth rate of shrimp is associated with disease outbreaks among commercially reared shrimp, it was important to assess the impact of replacing FM with YM on the shrimp immune system. We challenged shrimp after an 8-week feeding trial with an injection of *V. parahaemolyticus*—a pathogenic bacteria that is known to lead to high mortality among shrimp. We found that during the early development and growth stages of shrimp, YM could favorably replace FM in isoproteic and isoenergetic diets, with positive effects on growth performance and shrimp immunity.

### 4.1. Growth Performance

Replacing FM with YM improved all of the growth performance parameters of shrimp (see Table 3). When compared with the control diet based on FM only, specific growth rates were significantly higher among shrimp that were fed diets in which 25%, 50%, and 100% of FM were replaced with YM. Moreover, replacing from 25% to 75% of FM with YM led to significant increases in weight gain (up to +34%). Choi et al. (2018) reported similar results when replacing FM with a full-fat *T.molitor* meal in Pacific white shrimp diet. The authors suggested that an insect meal improves growth performance of the shrimp because it contains essential amino acids [21].

In 2019, Ido et al. reported significant improvements in the growth performances of red seabream (*Pargus Major*) with the gradual inclusion of up to 65% defatted insect meal in seabream diets [34]. In that study, the weight gain in seabream gradually increased; up to 77.8% when all FM was replaced by a full-fat *T.molitor* meal.

To date, various studies have confirmed high proportions of crude protein in insect meals [12,13,20], and a high content of essential amino acids with an adequate amino acid profile [35,36,37,38] except for the methionine and lysine levels, which should be supplemented to match the amino acid requirements of shrimp when the animal protein meal inclusion is limited to 20–30%. For instance, Panini et al. (2017) did not find improvements in Pacific white shrimp growth performance when using diets with non-defatted *T. molitor* meal without the supplementation of these amino acids [20].

In the present study, the inclusion of YM in experimental diets did not impact on the feed intake. It is interesting to note that in other nutritional studies, feed consumption was also not affected by dietary inclusion of mealworm protein [20,21].

As with other feed performance parameters, the FCR (Table 3) followed a significant quadratic pattern in relation to FM replacement with a minimal value obtained for a 50% FM replacement. The FCR was significantly improved by 0.385 points for the 50% FM replacement diet (FCR = 1.203) in comparison to the control diet (FCR = 1.588).

Considering these results, YM could be a good candidate as an alternative protein source for replacing FM in Pacific white shrimp diets. Indeed, most other protein sources tested to date as dietary FM substitutes, do not allow a complete replacement of FM, except for the vegetable protein mixture proposed by Amaya et al. (2007) [4] (gradual inclusion of soybean meal and corn gluten meal in combination with poultry by-product meal). In that study, replacing all FM with a vegetable and animal protein mixture did not negatively affect the growth parameters of Pacific white shrimp. All other vegetable-based alternative protein sources tested to date, lead to decreases in growth or feed utilization when FM is completely substituted [4,39,40]. Similarly, diets using single-cell protein from *Corynebacterium ammoniagenes* culture [41] or microalgal biomass from *Spirulina* (*Arthrospira platensis*) and *Nannochloropsis oculata* [42] lead to slower growth rates in Pacific white shrimp with increasing FM replacement, limiting the maximum inclusion of single-cell protein and microalgae to 20% and 50%, respectively. Considering animal proteins, fish blood by-products have also been tested [43,44], but the results were discouraging because it led to a decrease in growth performance, only permitting a maximum of 4% of FM replacement.

Considering insect protein as FM replacement, a potential insect candidate for large scale production as aquaculture feed [11,19], is the black soldier fly. However, in a study using black soldier fly larvae (*H. illucens*), FM replacement was limited to less than 25% of a diet for rainbow trout [18]. In contrast, all studies (including the present one) that used *T. molitor* as a dietary ingredient could completely replace FM in Pacific white shrimp diets [20,21], and some levels of FM substitution could even increase growth and feed conversion performances [21]. This was not the case in the study by Panini et al. (2017) and we propose that the negative impact on nutrient digestibility, observed in the Panini study, was either due to the absence of processing of their meal and absence of amino acid supplementation (methionine and lysine) rather than to the presence of chitin, as the authors suggested. The process quality (defattening and drying) of the *T. molitor* meal and the adequate amino acid supplementation seemed to be the main factors responsible for the improvement of shrimp growth performance. YM is higher in crude protein content than black soldier fly-based meals (70–74.8% versus 55% in crude protein, respectively). Insect protein meals contain nucleotides, hydrosoluble and insoluble proteins, bioactive peptides, vitamins, chitin, fatty acids, and other nutrients. The YM from Ÿnsect has a 20% hydrosoluble protein content over the total protein content with a high proportion of peptides (Table S1 of Supplementary Material). The nucleotide composition reaches 2.9 g/kg (dry matter) (Table S2 of Supplementary Material). Moreover, it is acknowledged that the nutrient composition, the drying process, the freshness of animal protein included in the preparation, and the process quality can lead to different digestibility and nutritional value for similar animal protein sources [17]. This highlights the need to include nutrient digestibility studies in future research of growth performance.

### 4.2. Immune Parameters

There is also interest in identifying a dietary source that would improve the immunity of shrimp so as to limit the losses due to pathogenic infection.

In our study, the total hemolymph protein concentration of pre-challenged shrimp tended to increase with increasing dietary levels of YM. Following the immune challenge with *V. parahaemolyticus,* the hemolymph protein decreased with increasing dietary YM levels. It is not known which of the proteins were affected in the present study. Zhu et al. (2018) showed that circulating proteins in shrimp hemolymph under normal conditions, are involved in five types of biological processes: binding proteins, proteins involved in the catalytic, enzymatic, regulatory, structural molecule, and translation regulator activity [45]. Nonetheless, proteomic studies have shown that in shrimp infected with bacteria, the changes of traditionally recognized immune-related proteins that are associated with the pro-PO system during phagocytosis, mostly act on the cytoskeletal level [46]. The reduction of the protein concentration in bacterial-challenged shrimp is not necessarily detrimental; a reduction of allergy-related proteins, for example, would be a potential positive effect of insect meal containing diets. However, further studies are required to assess which types of proteins are affected during bacterial challenge of insect meal fed shrimp.

Choi et al. (2018) reported significant improvements in the THC when insect meal fed shrimp (50% FM replacement) were challenged with the pathogen that causes white spot syndrome virus (WSSV) [21]. Similarly, in the present study, in pre-challenged shrimps, THC showed an improvement that positively correlated with the YM dietary levels. As expected, the bacterial challenge reduced the THC compared to the values obtained in shrimps not exposed to the bacteria. However, THC still increased in a dose-dependent manner with increasing dietary YM content after the challenge, suggesting that YM inclusion protected the shrimp against the observed disease-induced immunosuppression.

Concerning PO activity; it is well known that the PO system is the most important immune system in crustaceans [47,48,49]. In our study, at the end of the feeding trial, we observed a significant increase in PO activity with increasing levels of dietary YM. A strong immunosuppression was observed after the bacterial challenge in all of the tested shrimp groups. The PO response was reduced on average by 60% in bacterially challenged shrimp compared to pre-challenge shrimp. After the bacterial challenge, the PO activity of shrimps fed YM was as much as 2.5 times higher than that of shrimps fed FM.

We suggest that the infection-triggered reduction of the PO activity was likely due to the utilization of the enzyme to fight the infection. However, the PO level remained higher in the YM-fed shrimp. Furthermore, shrimp pathogen clearance ability, tested 3h after the injection challenge, improved as the dietary levels of YM increased; there were significantly less persisting bacteria in the hemolymph of shrimp fed higher levels of YM.

Our results suggest that the YM-modulated immunostimulation may well improve the ability of shrimp to clear bacterial pathogens from their circulatory system. Previous challenge tests have shown the presence of *V. parahaemolyticus* in the hemolymph from 30 min up to 6 h after the onset of the bacterial challenge [49,50].

We propose that the improvements in immunity and the enhanced ability to clear pathogens lead to a more than 60% reduction in shrimp mortality for shrimp fed diets in which >50% FM was replaced by YM versus those who received FM only. In contrast, in previous trials with shrimp, there were no significant reductions in the cumulative mortality [21]. Our results show that the inclusion of YM in shrimp diets can have a robust effect on the survival of shrimp after a bacterial challenge and that dietary YM could have immunostimulating benefits and in this way enhance disease resistance. We propose that the immunological benefits of YM are due to one or a combination of components such as chitin, polysaccharides, nucleotides, antimicrobial peptides, and/or melanin pigments–all of which are present in invertebrates [51,52,53,54,55,56]. Chitin has been shown to be immunostimulating for both shrimps and fish [27,57,58,59,60]. An in vitro digestibility study showed that chitin represents 5.75% of the YM [44]. Therefore, the YM containing diets used in the present study contained between 0.3% and 1.17% of chitin. There is evidence that chitin acts as an immunomodulator and it can, therefore, improve the survival rate of crustaceans exposed to diseases and to low dissolved oxygen stress [58,59]. Other potential immunostimulating components that may be present in YM, include polysaccharides such as silkrose or dipterose, isolated from silkworm and melon fly, respectively. These have been shown to be immunostimulating not only in mammals [55,56] but also in penaeid shrimp [61]. Although not yet demonstrated in mealworm, the presence in YM of such polysaccharides or of the other bioactive substances mentioned before (nucleotides, antimicrobial peptides, melanin pigments) could explain some of the immune protection against bacterial infection observed in the present study. Further studies will be necessary to determine which immunomodulating components of YM are responsible for the beneficial effects observed in our study.

In fish, mealworm meal-based diets have been shown to reduce the oxidative stress and inflammatory responses (ceruloplasmin, myeloperoxidase, and nitric oxide) [62]. In European seabass, mealworm meal enhanced both lysozyme antibacterial activity and serum trypsin inhibition, which are linked to antibacterial and antiparasitic activities respectively [62]. Krill meal-based diets have been shown to improve gut microbiota diversity and immunity of fish [28]. Similarly, full-fat mealworm meal diets change the microbiota in trout, seabream, and seabass. The microbiota in each of the fish species was differently affected by the inclusion of insect meal [63] and/or by the fat composition [63,64]. Researchers have hypothesized that the dietary effects of defatted insect meal-based diet on the gut microbiota of shrimp and the gene expression of the innate immune system, improve immunity and growth performance in shrimp [21,65].

## 5. Conclusions

Our study supports the inclusion of ŸnMeal^TM^ in shrimp diets, as it substantially improves growth when combined with FM without causing adverse effects on shrimp survival or feed ingestion even when 100% of FM is replaced by YM. Furthermore, one of the most important benefits of this feed ingredient is the improvement of immunity and resistance to disease, which translates to an improvement of shrimp survival. Improvement of immunity is of great importance as the intensity of shrimp production increases with concomitant stress and increased sensitivity to diseases. Our results confirm the great potential of ŸnMeal^TM^ (YM) as a functional feed source for farmed shrimp.

## Figures and Tables

**Table 1:** Yellow mealworm meal (ŸnMeal^TM^) proximate composition (% Dry Weight).

Nutritional Composition	
Moisture (%)	3.9
Crude Protein (%)	74.8
Crude Fat (%)	12.6
Crude Fiber (%)	4.7
Ash (%)	2.8

**Table 2:** Experimental diets formulation and proximate composition (% Dry Weight).

Experimental Diets Fish Meal Replacement (%)	Control 0%	YM25 25%	YM50 50%	YM75 75%	YM100 100%
**Ingredients**					
Fish meal 61% CP, Tuna	25	18.75	12.5	6.25	0
Soybean	25	25	25	25	25
Wheat gluten	10	10	10	10	10
Squid liver powder	5	5	5	5	5
Wheat flour	26.7	26.55	26.65	26.15	27.15
Yellow Mealworm meal	0	5.2	10.3	15.4	20.5
Tuna fish oil	2	2	2	2	2
Soya oil	1	0.85	0.75	0.65	0.55
Soy lecithin	2	2	2	2	2
Lysine	0	0.1	0.2	0.3	0.4
Methionine	0	0.05	0.1	0.15	0.2
Monocalcium phosphate	0	1.2	2.2	3.3	4.5
Lime stone/oyster shell	0	0	0	0.5	1
Binder	1.7	1.7	1.7	1.7	1.7
Vitamin premix *	1.6	1.6	1.6	1.6	1.6
**Sum**	100.00	100.00	100.00	100.00	100.00
**Proximate composition (AOAC, 2000)**					
Moisture (%)	9.38	8.82	8.75	9.02	9.45
Protein (%)	35.30	35.66	35.73	36.39	36.37
Lipid (%)	7.18	7.16	7.18	7.06	7.06
Fiber (%)	2.86	2.86	2.85	2.79	2.78
Ash (%)	8.25	8.02	7.35	6.65	6.03
Energy (MJ/kg)	18.72	18.56	18.45	18.27	18.09

* Composition of the Vitamin premix: Choline chloride (50%) 0.3%, StayC 0.5%, DSM2050 0.2%, anti-oxidant 0.1%, Anti-mold 0.1%, Mold inhibitor 0.2%, Diluent 0.2%; AOAC: Association of Official Agricultural Chemists.

**Table 3:** Growth, feed intake, and use, as well as mortality rates, among different fishmeal replacement diets (n = 6).

Experimental Diet	Control	YM25	YM50	YM75	YM100	*p*-Value		
Fish meal replacement (%)	0	25	50	75	100	L	Q	C
Final biomass (g/tank)	63.767a (3.192)	72.217ab (4.123)	79.0b (3.598)	78.55b (2.861)	71.617ab (3.067)	0.047	0.005	0.652
Initial body weight (g/ind)	1.60a (0.051)	1.50a (0.090)	1.60a (0.042)	1.66a (0.052)	1.46a (0.025)	0.481	0.250	0.012
Final body weight (g/ind)	5.542a (0.166)	6.432ab (0.435)	6.871b (0.291)	6.649b (0.238)	6.226ab (0.219)	0.085	0.004	0.780
Weight gain (g/ind)	3.940a (0.168)	4.933b (0.401)	5.271b (0.276)	4.986b (0.232)	4.768ab (0.213)	0.052	0.005	0.396
ADG (g/ind/day)	0.07a (0.003)	0.088b (0.007)	0.094b (0.005)	0.089b (0.004)	0.085ab (0.004)	0.052	0.005	0.396
SGR (%/d)	2.217a (0.07)	2.593b (0.129)	2.597b (0.072)	2.473ab (0.07)	2.588b (0.058)	0.025	0.046	0.027
Daily feed intake (g/ind/d)	0.11a (0.006)	0.114a (0.008)	0.112a (0.005)	0.115a (0.005)	0.112a (0.005)	0.807	0.696	0.993
PER	1.796a (0.161)	2.135a (0.148)	2.328a (0.143)	2.117a (0.105)	2.057a (0.08)	0.230	0.020	0.475
FCR	1.588b (0.13)	1.32ab (0.088)	1.203a (0.076)	1.304ab (0.07)	1.321ab (0.052)	0.051	0.020	0.390
Mortality rate (%)	0.233a (0.045)	0.244a (0.0453)	0.233a (0.0446)	0.211a (0.043)	0.233a (0.0446)	0.812	0.943	0.635

The data means with the same letters within rows were not significantly different (*p* > 0.05.) The data’ standard error of the mean (SEM) are presented in parentheses. L, Q, and C stand for linear, quadratic, and cubic contrasts, respectively. ADG: average daily gain; SGR: specific growth rate; FCR: feed conversion ratio; PER: protein efficiency ratio.

**Table 4:** Mortality as a function of dietary insect meal (YM) levels.

Variable	Coefficient	SE	Df	$\chi^{2}$	$\mathrm{Pr}\;(>\chi^{2})$
Mortality					
Intercept	−0.213	0.353	-	-	
Dietary YM Level	−0.041	0.019	1	4.585	0.032
Dietary YM Level^2^	2.53 × 10^−4^	1.97 × 10^−4^	1	1.634	0.201

Generalized linear model with binomial error distribution and logit link; response equation: $p\left(x\right)=\frac{\;e^{\alpha +\beta_{1}DYML+\beta_{2}DYML^{2}}}{\;1+e^{\alpha +\beta_{1}DYML+\beta_{2}DYML^{2}}}$; *p* = cumulative probability of dying; Dietary YM Level (DYML) = Percentage of FM replaced by Ynsect Meal; $\chi^{2}$ test statistic for Likelihood-ratio test.

**Table 5:** Different immunological responses as a function of bacterial challenge status and dietary YM level.

Variable	Coefficient	SE	Df	$\chi^{2}$	$\mathrm{Pr}\;(>\chi^{2})$
**Phenoloxidase (Units/mL)**					
Intercept	5248	673.9	-		
Dietary YM Level	27.4	11.0	1	53.883	2.128 ×10^−13^
Status: challenged	−3735	819.5	1	70.104	2.2 × 10^−16^
Dietary YM Level × Status: challenged	−4.5	13.4	1	0.115	0.7348
**Hemocyte count (x10^6^ cells)**					
Intercept	1.073	0.064	-		
Dietary YM Level	0.003	0.001	1	14.136	1.7 × 10^−4^
Status: challenged	−0.088	0.021	1	117.933	2.2 × 10^−16^
Dietary YM Level x Status: challenged	−0.001	3.5 × 10^−4^	1	6.949	8.4 × 10^−3^
**Hemolymph protein (g/dL)**					
Intercept	4.796	0.668	-		
Dietary YM Level	0.014	0.011	1	4.832	2.8 × 10^−2^
Status: challenged	1.373	0.559	1	3.054	8.1 × 10^−2^
Dietary YM Level x Status: challenged	−0.039	0.009	1	18.018	2.2 × 10^−5^
**Bacterial persistence (log cfu/mL)**					
Intercept	4.359	0.058	-		
Dietary YM Level	−0.007	0.001	1	47.865	4.6 × 10^−12^

Generalized Least Squares Model:$\;Immunological\;response=\;\alpha +\beta_{1}DYML+\beta_{2}Status\;+\;\beta_{3}DYML\;\times \;Status$; Status: challenged (1) and pre-challenged (0). Dietary YM Level (DYML) = Percentage of FM replaced by Ynsect Meal; $\chi^{2}$ Test statistic for Type II Wald Chi-square test.

## Supplementary Materials

The following are available online at https://www.mdpi.com/2076-2615/9/5/258/s1, Figure S1: Comparison of cumulative mortality (%) per dietary treatment 10 days after the bacterial challenge (*n* = 30). Different letters show significant differences between dietary groups. Figure S2: Immune status of the shrimp before and after bacterial challenge with PO expressed as units/mg of protein. A) Phenoloxidase B) hemocyte counts C) hemolymph protein D) persistent bacterial numbers in hemolymph 3h after the bacterial challenge. Confidence bands for each fitted line correspond to one SEM (n = 3). Table S1: Ÿnsect meal of defatted yellow protein; hydrosoluble part and distribution size of protein of the hydrosoluble part. Table S2: Nucleotide composition of Ÿnsect meal.
