# Supplementary material for: Replacing Fish Meal with Defatted Insect Meal (Yellow Mealworm Tenebrio molitor) Improves the Growth and Immunity of Pacific White Shrimp (Litopenaeus vannamei)
Source: Animals (Basel). 2019 May 21;9(5):258. doi: 10.3390/ani9050258 (PMC6563494; doi:10.3390/ani9050258)
Supplement: Supplementary file 1 [file animals-09-00258-s001.pdf]

animals

MDPI

Supplementary Materials: Replacing fish meal with defatted insect meal (yellow mealworm *Tenebrio molitor*) improves the growth and immunity of Pacific white shrimp (*Litopenaeus vannamei*)

Table S1. Ÿnsect meal of defatted yellow protein; hydrosoluble part and distribution size of protein of the hydrosoluble part.

Size (kDa)	Percentage (%)
Soluble protein	20,02
<0,555	68,54
1,4–0,555	4,45
6,5–1,4	5,89
12,4–6,5	11,56
>12,4	9,57
insoluble protein	79,98

Table S2. Nucleotide composition of Ÿnsect meal.

Nucleotide	Ÿnsect defatted meal (g/kg)
Cytosine	0,025
Cytidine	0,185
Uracyl	0,013
Guanine	0,1
Uridine	0,478
Hypoxanthine	0
Adenine	0,165
Guanosine	0
Adenosine	1,467
Inosine	0
Cytidine Mono-Phosphate	0
Uridine Mono-Phosphate	0,23
Gunaosine Mono-Phosphate	0,037
Iinosine Mono-Phosphate	0,04
Adenosine Mono-Phosphate	0,175
**TOTAL**	**2,915**

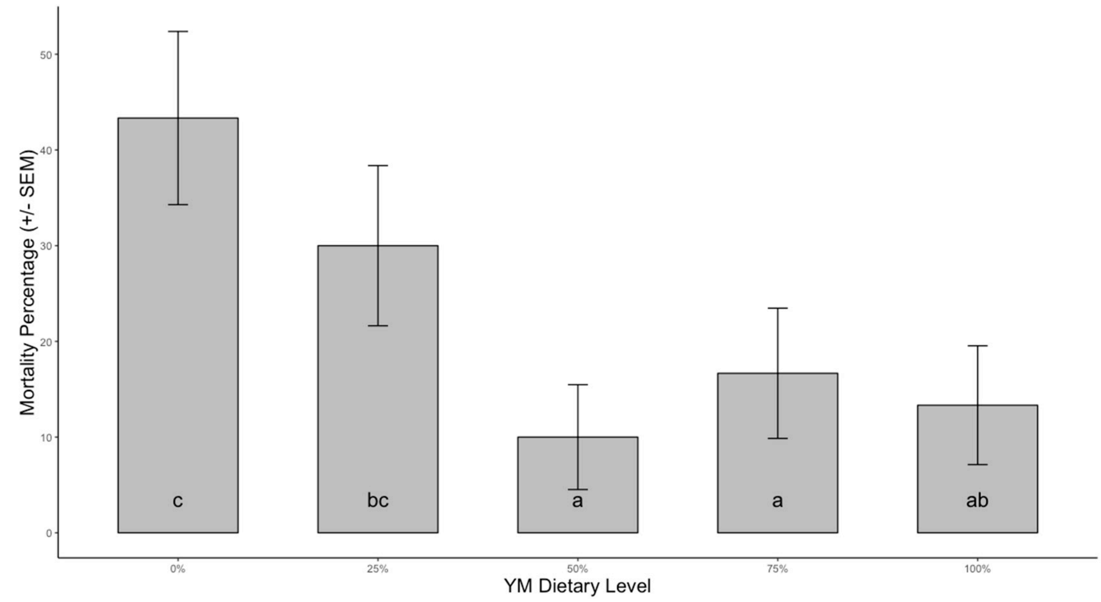

Figure S1: Comparison of cumulative mortality (%) per dietary treatment 10 days after the bacterial challenge (n = 30). Different letters show significant differences between dietary groups.

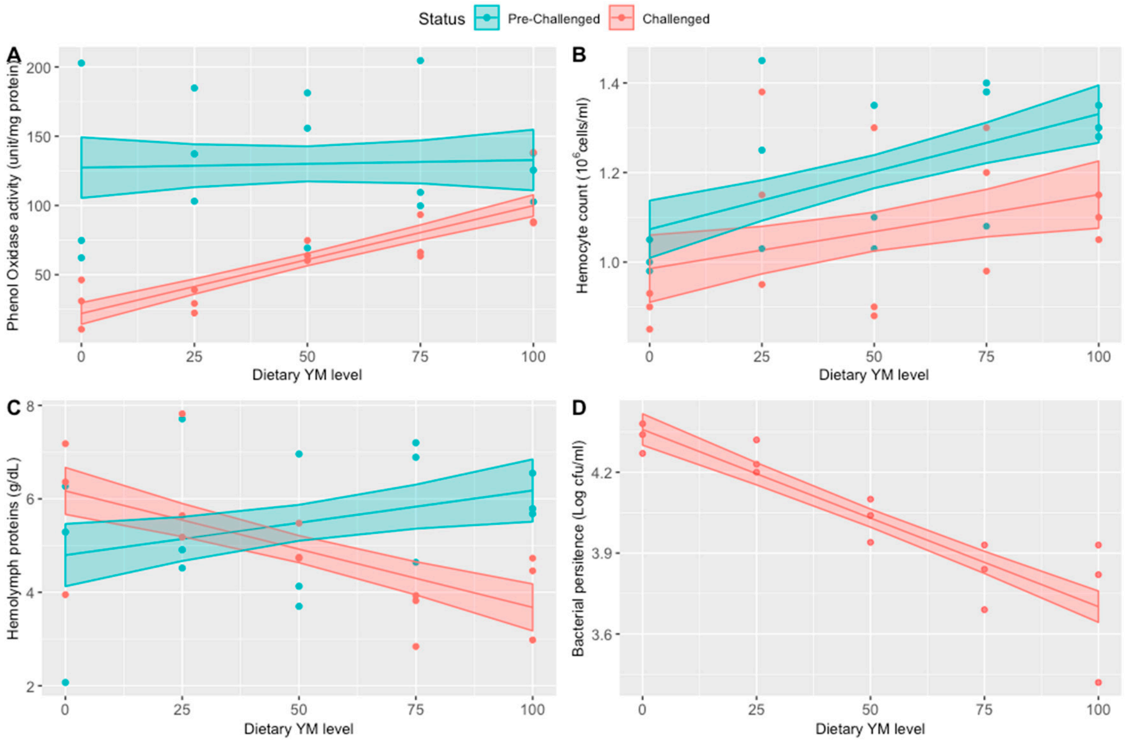

Figure S2. Immune status of the shrimp before and after bacterial challenge with PO expressed as units/mg of protein. (**A**) Phenoloxidase (B) hemocyte counts (**C**) hemolymph protein (**D**) persistent bacterial numbers in hemolymph 3h after the bacterial challenge. Confidence bands for each fitted line correspond to one SEM (n = 3).

© 2019 by the authors. Licensee MDPI, Basel, Switzerland. This article is an open access article distributed under the terms and conditions of the Creative Commons Attribution (CC BY) license (http://creativecommons.org/licenses/by/4.0/).
